# Editorial: Towards genome interpretation: Computational methods to model the genotype-phenotype relationship

**DOI:** 10.3389/fbinf.2022.1098941

**Published:** 2022-11-30

**Authors:** Daniele Raimondi, Gabriele Orlando, Nora Verplaetse, Piero Fariselli, Yves Moreau

**Affiliations:** ^1^ ESAT-STADIUS, KU Leuven Leuven, Belgium; ^2^ Switch Laboratory, KU Leuven Leuven, Belgium; ^3^ Department of Medical Sciences, University of Torino, Torino, Italy

Genome Interpretation (GI) is an umbrella term for the scientific efforts oriented towards modelling and understanding the relationship between genotype and phenotype in living organisms (Daneshjou et al., 2017; Andreoletti et al., 2019; Raimondi et al., 2022a). Even temporarily setting epigenetic and environmental effects aside, untangling the complex relation between the complete set of genetic material of an individual organism (be it a human, other animals, plants, or microorganisms) and its observed phenotypes is an extremely ambitious and challenging endeavor, in particular for non-Mendelian traits. Being able to reliably model this genotype-phenotype relationship could revolutionize many aspects of genetics, biology, and medicine (Daneshjou et al., 2017; Fröhlich et al., 2018). For example, it could warn us about late-onset genetic disorders, helping their prevention (Weedon et al., 2006; Morrison et al., 2007). It could also lead to the design of medications and treatments tailored to each patient’s genome, complementing environmental and medical-history data to improve patient prognosis (Fröhlich et al., 2018). Applied to cancer, it could bring a novel understanding of cancer development, helping devise highly specific cocktails of drugs and discover novel molecules to target each unique tumor (Li et al., 2019). Such personalized approaches to medicine, called Precision Medicine, are still largely out of our reach in many clinical settings (Daneshjou et al., 2017; Fröhlich et al., 2018).

In the last decade, the avalanche of scientific results brought by Next Generation Sequencing (NGS) and big data technologies seemed almost unstoppable, and at times it seemed that finally *cracking* the genotype-phenotype problem was within reach. Ten years later, notwithstanding the vast amounts of data collected and numerous advances in genetics (Moreau and Tranchevent, 2012; Boycott et al., 2013; Erwin et al., 2014; Goodwin et al., 2016), including the discovery of the causative variants for many Mendelian disorders (Bamshad et al., 2011), our genome is still hiding most of its secrets. When it comes to oligogenic and polygenic diseases (i.e., diseases involving respectively few and many genes (Gazzo et al., 2017)), the bottleneck has indeed mostly just shifted from a problem of data availability to one of data interpretation, since the classical approaches used in genetics have shown important shortcomings in uncovering complex disease mechanisms (Manolio et al., 2009; Gibson, 2012; Francisco and Bustamante, 2018; Wald and Robert, 2019).

The advent of NGS technologies was nonetheless invaluable, since they almost brought us at the doorstep of a new era where the scarcity of genomics data will be less and less of a bottleneck. This will make the application of *data hungry* cutting-edge Machine Learning (ML) and Deep Learning (DL) methods to this endeavor finally possible, eventually reproducing the astounding successes that methods such AlphaFold (Jumper et al., 2021; Chowdhury et al., 2022) obtained in structural biology in the realm of Genome Interpretation.

However, data abundance alone will not do the trick, for such a complex problem. The actual implementation of ML/DL methods for GI requires the development of tailor-made algorithms that can deal with the unique issues presented by genomic and phenomics (Houle et al, 2010) data. For example, Whole Exome or Genome Sequencing samples (WES, WGS) can be extremely large, sparse, and noisy (Ng et al., 2008). Moreover, they also pose privacy and ethical issues in their management, storage, and processing (Rieke et al., 2020). Finally, to apply GI to Precision Medicine, models must ensure *accountability* of their predictions, for example by providing means for their interpretability and explainability, following the Explainable AI (XAI) paradigm (Bach et al., 2015; Smilkov et al., 2017; Lapuschkin et al., 2019; Raimondi et al., 2020a).

In the last decade, the bioinformatics community has addressed various specific aspects related to the Genome Interpretation (GI) problem, developing variant-effect predictors (Kircher et al., 2014; Dong et al., 2015; Ioannidis et al., 2016; Jagadeesh et al., 2016; Niroula and Vihinen, 2016; Raimondi et al., 2016; Raimondi et al., 2017), variant-prioritization (Sifrim et al., 2013; Wu et al., 2014; Cipriani et al., 2020) and gene-prioritization tools (Aerts et al., 2006; Guala and Sonnhammer, 2017), also trying to model digenic disease (Gazzo et al., 2017; Papadimitriou et al., 2019) or the protein-level molecular phenotype caused by a variant (Dehouck et al., 2011; Pucci et al., 2020; Raimondi et al., 2022b). Other widespread approaches in this sense include Genome Wide Association Studies (GWAS) (Uffelmann et al., 2021) and Polygenic Risk Scores (PRS) (Wei et al., 2013; Ali et al., 2018; Ala-Korpela and Holmes, 2020; Badré et al., 2021). In the context of plant and animal sciences, genetic marker-based methods for the Genomic Prediction for plants and animal breeding (e.g., BLUP) have been widely used (Daetwyler et al., 2013; Hickey et al., 2017; Wray et al., 2019; Maldonado et al., 2020).

These methods are the most relevant examples of how GI has been tackled so far. Few of them aim at directly modeling the genotype-phenotype relationship, while most focus instead on simpler subproblems, such as predicting the neutral/deleterious effect of variants or just finding associations between phenotypes and genomic regions.

The growing availability of genomics data will soon enable the application of the latest ML/DL algorithms to GI, attempting to directly model the phenotypes produced by a given genome or exome, following a “genomes in/phenotypes out” paradigm (Raimondi et al., 2020b; Raimondi et al., 2022a). Early examples of such an approach, although on limited data, are methods for the case-controls discrimination of Crohn’s Disease (Wang et al., 2019; Raimondi et al., 2020b), Bipolar Disorder (Laksshman et al., 2017), the multi-phenotypic prediction of *A. thaliana* (Raimondi et al., 2022a) and yeast quantitative traits (Grinberg et al., 2020). We can imagine these methods as framed within a *spectrum of complexity*: at the *narrow end* of the spectrum we have methods aiming at the binary prediction or regression of the presence/absence of a certain phenotype (e.g., in cases/control studies) (Pal et al., 2017; Raimondi et al., 2020b), while at the *broad end* of the spectrum we have methods that perform a multiphenotypic prediction given a certain type of genotype measurement (e.g., WES, WGS or SNP array data) (Grinberg et al., 2020; Raimondi et al., 2022a).

In this Research Topic we collect papers that develop computational and ML methods addressing the challenges posed by this new paradigm of ML-based GI. These studies range from the application of GI to prokaryotes, with methods for the identification of putative cellulolytic anaerobes and for the identification of microsatellites that could act as biomarkers to differentiate *C. pseudotuberculosis* genomes, to yeast, with a Sparse Bayesian method for the prediction of *S. cerevisiae* growth in 46 different environmental conditions. Finally, regarding the development of strategies to apply DL methods to GI in the future, we propose a study investigating the possibility of encoding human genotype data as images, thus making it suitable for the application of DL techniques such as Convolutional Neural Networks for case/control classification.

While this Research Topic is by no means conclusive for a complex and long-term problem such as GI, we hope it can help focus more debate and research efforts on this *flavor* of ML/DL based GI, paving the way for full-fledged applications of this paradigm once large-scale genomic and phenomic data become widely available.
